# Consumption in Illinois

**Published:** 1844-12-28

**Authors:** 


CONSUMPTION IN ILLINOIS.

For the last fortnight, during which I have conversed with the physicians of seven towns between Jacksonville and Joliet, I have been assured, *nemine contradicente*, that tubercular consumption is one of the rarest diseases of that part of Illinois which lies between St. Louis and Chicago. Most of them have not seen an indigenous case, and all have known of immigrants in whom the progress of the disease, in its early stage, had been arrested or retarded. As many of our readers may be consulted by their patients, concerning the effect of the climate of this region on those inclined to phthisis, I have thought it proper to anticipate a future publication by this brief notice.—*Dr. Drake, in Western Journal.*

